# Fellow-Workers and Their Doings

**Published:** 1914-06-20

**Authors:** 


### ROUND THE WORLD.

### Fellow-Workers and their Doings.

[The Editor Invites Early Information and Contributions Marked "Fellow-Workers."]

Mr. Alexander G. R. Foulerton, who has just been appointed lecturer in the public health department at University College, London, is well known to Middlesex Hospital men as the director of the bacteriological and clinical pathological laboratories at that institution. Dr. Foulerton was a student at "Bart.'s"; qualifying in 1884, he became an F.R.C.S. in 1895 and D.P.H.Eng. in 1892. Formerly he was director of the Cancer Research Laboratory and assistant bacteriologist at the Lister Institute, whilst his present appointments include that of examiner in hygiene at the Royal Army Medical College. He is also well known as Financial Secretary to the London and Counties Medical Protection Association.

Some very interesting observations on the cardiac complications of acute rheumatism in childhood have lately been made by Dr. G. A. Sutherland, M.D., F.R.C.P., who has recently brought before the notice of the Royal Society of Medicine a series of cases illustrating his views. One of the most important points emphasised by this observer is that in children suffering from rheumatism affecting the heart the condition known as "auricular flutter" is far more commonly present than is usually supposed. The result of this is said to be that a great deal of strain is thrown on the ventricles through back pressure, and the heart, already weakened by the carditis, may rapidly fail. Dr. Sutherland is one of the physicians to the Paddington Green Children's Hospital.

Dr. T. H. Oliver, who has just been appointed surgical registrar to the Manchester Royal Infirmary, studied medicine both there and at Cambridge. Qualifying M.B., Ch.B. Vict.Manch., with honours, some two years ago, Dr. Oliver subsequently held important resident appointments at the institution where he is now being again elected a member of the staff.

In these days, when the knife appears to be relied upon more and more each year for the relief of various ailments, it is interesting to note that an expert of experience has been entering a plea for fewer operations in the treatment of tonsils and adenoids. Mr. T. Bramley Layton, M.S., F.R.C.S., of the throat and ear department at Guy's Hospital, has lately been teaching that there are important considerations in favour of preserving the tonsils whenever possible, as he maintains that these organs are an important feature of our defence against microbic invasion. From the point of view of treatment, this authority considers that attention to general health, breathing exercises, and the care of the teeth will go far to remedy many cases for which operation is now recommended. Besides being in charge of the throat and ear department at Guy's, Mr. Layton, who won the gold medal at the M.S. London examination in 1907, is senior demonstrator in anatomy in the medical school.

Dr. Frederick St. John Kemm, who has just given up his work as medical officer for the Worle district, in Somersetshire, has served the local board of guardians for over thirty years, during which long period he has been assiduous in his attention to the poor invalids coming under his care. Dr. Kemm qualified from the London Hospital in 1881, and became an M.D. Durham in 1904.

It is likely that the next great advance in radiography will come from work that is now being done on the filtration of *x*-rays. Much important investigation is being carried out in various laboratories with the object of finding out the best substances to use as filters, so that it is possible in the near future an apparatus may be constructed which will enable the maximum dose of *x*-rays to be given with the minimum danger. Dr. R. W. Salmond, M.D. Aberd., is one of the workers whose researches have lately done much to increase our knowledge of *x*-ray filtration, and, although he considers that radiologists have several excellent filters at their disposal to-day, he is of opinion that the ideal filter has yet to be found. Dr. Salmond, who came to London from Aberdeen, has been carrying out these investigations at the electro-therapeutic department at the Cancer Hospital; he is assistant radiographer to King's College Hospital and surgeon-in-charge of the *x*-ray department at St. Mary's Hospital, Plaistow.

The new pathologist to the St. Mary's Hospitals, Manchester, Dr. Daniel Dougal, is an Owens College student, and was awarded the gold medal at the M.D. examination of the Victoria University at Manchester last year. Latterly he has been resident obstetric officer to the St. Mary's Hospitals.

The technique of spinal analgesia owes much to the keenness with which Mr. Lawrie McGavin, F.R.C.S., has followed up the method. By dint of perseverance this well-known surgeon seems to have found out just what combination of drugs and positions of the patient will give the best results, and how to avoid the unpleasant after-consequences, particularly headache, which affects some who have been anæsthetised in this way. Many of his recent observations have been published in the *Clinical Journal*; in reporting them he has called attention to the constancy with which a rise of temperature from 99° to 101° follows the injections. Mr. McGavin was in earlier years attached to Guy's Hospital, where, after qualifying, he held the positions of assistant demonstrator in anatomy and surgical registrar; subsequently he was assistant surgeon to the North-West London Hospital, and now holds the posts of surgeon to the Seamen's Hospital at Greenwich and to the Hospital for Women in Soho.

Dr. Lovell Drage continues to be interested in the chemical treatment of cancer, and in the *Lancet* (June 6) suggests that copper in the form of a protalbuminate or lysalbuminate is able to benefit patients suffering from that disease in inoperable form. The success of this method is said to be based on the fact that the copper compound in question presents the copper in colloidal form. Dr. Lovell Drage, who is an old "Bart.'s" man, has for some years been medical officer of health for the Hatfield district.

The vacancy for an assistant surgeon at the Cancer Hospital has been filled by the election of Mr. P. P. Cole, F.R.C.S., the registrar. Mr. Cole is an old Guy's man, and has been for some time assistant surgeon at the *Dreadnought* Hospital. He possesses the very unusual distinction, for a London surgeon, of a dental qualification, for he took the L.D.S. R.C.S., before deciding to take up the medical profession.

